# Successful Clozapine Rechallenge in a 40‐Year‐Old Male Patient With Paranoid Schizophrenia After Clozapine‐Associated Myocarditis

**DOI:** 10.1155/crps/3858403

**Published:** 2026-01-20

**Authors:** Lukas Zabel, Nathalie Ersek, Andreas B. Hofmann, Lena Machetanz, Johannes Kirchebner, Susanne Stübner

**Affiliations:** ^1^ Department of Forensic Psychiatry, University Hospital of Psychiatry Zurich, University of Zurich, Lenggstrasse 31, Zurich, 8032, Switzerland, uzh.ch; ^2^ Department of Adult Psychiatry and Psychotherapy, University Hospital of Psychiatry Zurich, University of Zurich, Zurich, 8032, Switzerland, uzh.ch; ^3^ Department of Psychiatry and Psychotherapy, Ludwig Maximilian University of Munich, Munich, Germany, uni-muenchen.de

**Keywords:** clozapine, clozapine-associated myocarditis, clozapine rechallenge, clozapine titration

## Abstract

**Background:**

Clozapine‐associated myocarditis (CAM) is the most common inflammatory adverse effect under clozapine treatment. In the absence of equally effective therapeutic alternatives for treating treatment‐resistant schizophrenia (TRS), clinicians are often faced with the question of whether to rechallenge clozapine in patients who have experienced CAM. However, there is a lack of standardized protocols and published case reports of successful clozapine rechallenge following CAM.

**Case Presentation:**

A 40‐year‐old patient diagnosed with paranoid schizophrenia experienced CAM during his first clozapine treatment. Four years later, he was successfully rechallenged using a recently published standardized protocol involving a very slow titration schedule.

**Conclusions:**

Our case adds to the limited number of published cases of successful clozapine rechallenge following CAM. It is the first published case report to use the rechallenge protocol recently published by Qubad et al. (2024), thereby contributing to the development of standardized protocols for the safe and successful clozapine rechallenge following CAM.

## 1. Introduction

Clozapine, a second‐generation antipsychotic agent, is best known for its superior efficacy in treatment‐resistant schizophrenia (TRS; e.g., [1]). However, it can be accompanied by adverse drug effects that can lead to serious or even fatal consequences, including clozapine‐induced agranulocytosis (CIA; [2, 3]) and inflammatory adverse effects such as myocarditis, nephritis or drug reaction with eosinophilia and systemic symptoms (DRESS; [4]). Clozapine‐associated myocarditis (CAM) is the most common of the inflammatory adverse effects with an incidence ranging from 0.03% to 8.5%, depending on factors such as titration speed, monitoring regimen, ethnicity, or metabolizer status [5, 6]. The mortality rate of CAM is estimated to be 4.8% [7]. The risk of developing CAM is highest during the first 4 weeks of treatment, particularly between Days 14 and 21 [8]. Clinical symptoms may be completely absent or range from nonspecific signs, such as fatigue or fever, to more specific signs, such as tachycardia, chest pain, or shortness of breath [6, 9]. CAM regularly leads to an elevation of inflammatory markers such as C‐reactive protein (CRP), cardiac troponin, or other cardiac markers [9]. Ronaldson et al. [8] found an elevation of cardiac troponin in 90% of cases. Although availability is limited, the gold standard for diagnosing CAM is cardiac biopsy or cardiac magnetic resonance imaging (CMR; [5]). Given the lack of equally effective alternatives to clozapine in the treatment of TRS, clinicians are often faced with the question of whether clozapine can be rechallenged in patients who have experienced CAM [6]. McMahon et al. [5] have stated that there is a lack of consensus regarding the process, monitoring, and dose titration to achieve a safe and successful clozapine rechallenge. In their recent review, they identified 45 cases of clozapine rechallenge following CAM, 31 of which (69%) were successful. In 14 cases (31%), the patients redeveloped CAM. Out of the 31 successful cases, only six cases followed published rechallenge protocols. Qubad et al. [6] recently published such a detailed instruction with a step‐by‐step guide and strong emphasis on the patient’s safety by proposing a very slow titration.

We present a case of a 40‐year‐old male patient diagnosed with TRS, who was successfully rechallenged with clozapine 4 years after developing CAM.

## 2. Case Presentation

A 36‐year‐old male patient of Kurdish origin was admitted to the high‐security ward of our institution (located in Switzerland) from a correctional facility. He had been diagnosed with paranoid schizophrenia 4 years ago. After being treated with different antipsychotics, including olanzapine, paliperidone, and cariprazine, in sufficient doses for at least 6 weeks without sufficient efficacy, he met the criteria for TRS [1] and was offered treatment with clozapine, to which he consented. The patient’s height was 172 cm and his weight was 70 kg (BMI: 23.7 kg/m^2^). The comedication was: quetiapine 200 mg, olanzapine 20 mg (stopped on Day 1), and cariprazine 3 mg (reduced to 1.5 mg on Day 5 and stopped on Day 10). The patient smoked 20 cigarettes a day.

### 2.1. First Clozapine Treatment

The first clozapine treatment, as will be described later, had several shortcomings. These shortcomings are easier to identify in retrospect. Rigorously assessing past cases provides an opportunity to learn from them and improve future treatment.

Before initiating clozapine, laboratory tests revealed an elevation of white blood cell (WBC) count (13.7 × 10^9^/L; normal range: 4.23–9.10 × 10^9^/L), neutrophils (8.08 × 10^9^/L; normal range: 1.56–6.13 × 10^9^/L), and eosinophils (0.89 × 10^9^/L; normal range: 0.04–0.54 × 10^9^/L). CRP and high‐sensitivity troponin T (hs‐TnT) were not assessed before initiation. Although there were no clinical signs of infection or inflammation, baseline inflammation should have been ruled out by assessing CRP and hs‐TnT, as proposed in international guidelines [10]. Starting clozapine treatment without this prior assessment posed an unnecessary risk, since systemic inflammation can impair clozapine metabolism, thereby increasing the risk of developing CAM. Baseline electrocardiogram (ECG) showed no abnormalities. Clozapine was dosed up to a dose of 100 mg by Day 4. This high titration speed was not justified by clinical factors (e.g., aggressive behavior or suicidality) and was much faster than that recommended by international guidelines, thereby posing an unnecessary risk of developing inflammatory adverse effects, including CAM [10]. On Day 4, the patient developed tachycardia (maximum 143 bpm) and nocturnal palpitations. In this situation, CRP and hs‐TnT should have been assessed to check for early signs of CAM. An ECG on Day 10 showed sinus tachycardia without other abnormalities. On Day 11, hs‐TnT was 1.5‐fold elevated (22 ng/L, normal range: below 14 ng/L), while CRP was not assessed. At this point, while taking a daily dose of 100 mg, the patient’s clozapine serum level (trough and steady‐state conditions) was 68 ng/mL. The clozapine concentration‐to‐dose ratio (clozapine C/D ratio) was 0.68, which is lower than would typically be expected for a male smoker of European or Western Asian ancestry [10]. The laboratory assay used was the kit (LC‐MS/MS) by Chromsystems; the result of the sample was available the following afternoon. However, repeat testing in the late afternoon on Day 11 showed normalization of hs‐TnT and other cardiac markers, such as creatine kinase (CK), CK‐myocardial band (CK‐MB), and myoglobin. Despite persistent tachycardia, as the patient showed no other clinical signs, it was decided to continue with a 100 mg dose of clozapine and start bisoprolol to control tachycardia. In retrospect, it would have been safer to stop clozapine and wait for evaluation by a cardiologist. On Day 12, the patient once experienced left‐sided stabbing chest pain. As the chest pain was most likely a symptom of CAM, it was a major mistake not to discontinue clozapine by this point. On Day 14, laboratory tests showed elevated WBC (12.6 × 10^9^/L; normal range: 4.23–9.10 × 10^9^/L), neutrophils (7.81 × 10^9^/L; normal range: 1.56–6.13× 10^9^/L), and eosinophils (1.20×10^9^/L; normal range: 0.04–0.54 × 10^9^/L); hs‐TnT was slightly elevated at 17 ng/L and CK‐MB was slightly elevated at 26 U/L (normal range: below 25 U/L). CRP was not assessed. On Day 16, clozapine was discontinued due to the laboratory abnormalities and chest pain. As transthoracic echocardiography (TTE) was unavailable at the psychiatric hospital, the patient was referred to the cardiology department of a nearby hospital, but due to scheduling delays during the Covid‐19 pandemic, the appointment was scheduled for December. After clozapine was discontinued, the tachycardia persisted (maximum 130 bpm) and the patient had another episode of chest pain on Day 43. Laboratory tests on Day 32 showed slightly elevated hs‐TnT (17 ng/L), which normalized by Day 58. On Day 89 (i.e., about 12 weeks after discontinuing clozapine), TTE revealed diffuse wall hypokinesia and mildly impaired systolic function with a left ventricular ejection fraction (LVEF) of 45%. CMR was recommended to confirm the diagnosis, but could not be performed due to the patient’s gunshot wound, which left countless metal fragments in the floor of his mouth. Cardioprotective therapy (perindopril, indapamide, bisoprolol, and aldactone) was initiated and well tolerated. On Day 148, TTE was repeated showing no more abnormalities and a LVEF of 55%. Although the first TTE was performed with a 3‐month delay, the case met the criteria for CAM according to Ronaldson et al. [11], as the patient developed clinical signs of dysfunction (tachycardia and chest pain) and diagnostic evidence of cardiac abnormalities (reduced LVEF and wall hypokinesia) within 45 days of clozapine initiation. The diagnostic criteria for clinically suspected myocarditis by the European Society of Cardiology [12] were also fulfilled.

After discontinuation of clozapine, the patient was treated with various combinations of antipsychotic medications, including quetiapine, cariprazine, and risperidone. For almost 2 years, his condition remained stable, allowing for significant progress that led to his transfer from a high‐security ward to a low‐security ward. Then, his condition began to deteriorate again: He became more psychotic, agitated, and aggressive, attacked fellow patients twice and had to be moved back to the high‐security ward.

Given the severity of his condition, a rechallenge with clozapine was then considered for the first time, but could not be further evaluated, because the patient, while treated with paliperidone 9 mg and quetiapine 600 mg, again experienced several episodes of left‐sided chest pain with laboratory studies showing elevations of hs‐TnT up to 40 ng/L, CRP up to 75 mg/L, WCC up to 18.5 × 10^9^/L, and neutrophils up to 13.32 × 10^9^/L. However, an ECG showed no abnormalities. Since the patient consistently refused to be transferred to a nearby hospital for cardiac evaluation including TTE, diagnostics necessary prior rechallenging clozapine could not be performed. After more than a year, he finally agreed to transfer and performing a TTE that showed no abnormalities with a normal LVEF of 62%.

### 2.2. Clozapine Rechallenge

Because of the patient’s ongoing treatment‐resistance with prominent positive and negative symptoms, the patient was offered a rechallenge with clozapine to which he consented. We followed a slightly adapted version of the rechallenge protocol recently published by Qubad et al. [6]: We strictly followed their instruction in terms of dose titration, but performed ECGs once instead of twice a week due to the patient’s request. As the patient initially declined to have blood samples taken twice a week, we also performed the laboratory assessment once per week for the first 4 weeks, after which we switched to twice a week. As the patient was being treated in an inpatient setting with the possibility of daily monitoring of clinical symptoms and signs, we accepted the patient’s request, despite presumably less safety. A baseline laboratory test showed WBC 9.7 × 10^9^/L (normal range: 3.9–10.2 × 10^9^/L), neutrophils 4.85 × 10^9^/L (normal range: 1.5–7.7 × 10^9^/L), eosinophils 0.63 × 10^9^/L (normal range: 0.02–0.5 × 10^9^/L), CRP 1.6 mg/L (normal range: <5 mg/L), and a slightly elevated hs‐TnT at 22 ng/L (normal range: <14 ng/L). However, in the 3 months prior to rechallenge, the patient’s baseline troponin levels were always up to 1.5‐times elevated. The troponin assay used was the Elecsys assay by Roche Diagnostics with high sensitivity and a threshold of 14 ng/L for males [13]. A baseline ECG showed no abnormalities. At the time of the clozapine rechallenge, the patient’s weight was 56 kg (BMI: 18.9 kg/m^2^). He smoked 20 cigarettes per day and did not change his smoking habits during titration. The comedication was: quetiapine retard 800 mg, amisulpride 800 mg, levomepromazine 120 mg, diazepam 10 mg, pantoprazole 20 mg, and ivrabadine 15 mg. Quetiapine, the coprescription of which may increase the risk of CAM [14], was gradually reduced and stopped after 19 weeks. Levomepromazine, which is possibly a mild inducer of CYP1A2 [10], was gradually reduced and stopped after 6 weeks. The amisulpride dose was reduced to 600 mg once the clozapine dose reached 400 mg. Bisoprolol 2.5 mg was added after 3 weeks to control tachycardia. It was not possible to stop diazepam beforehand, as recommended by international guidelines [10], since the patient did not tolerate any reduction, becoming more irritable and agitated.

Clozapine was started at dose of 6.25 mg and titrated up by 6.25 mg every 4 days (Table 1). After approximately 6 weeks, having reached a dose of 75 mg, the titration rate was increased to 6.25 mg every 3 days. After a further 6 weeks, having reached a dose of 150 mg, the titration rate was increased to 6.25 mg every 2 days. At the patient’s request, the full dose of clozapine was given once a day at night. Depending on plasma levels, clozapine was titrated up to a dose of 450 mg QHS, which was reached after approximately 26 weeks or half a year. At a daily dose of 450 mg, serum levels ranged from 278 to 435 ng/mL. All serum levels were measured under trough and steady‐state conditions, with the patient’s smoking habits and comedication remaining unchanged. Clozapine intake always took place under a nurse’s observation to ensure adherence. The mean clozapine C/D ratio, calculated from three plasma levels, was 0.75, which is lower than would typically be expected for a male smoker of European or Western Asian ancestry [10].

**Table 1:** Laboratory testing during clozapine titration.

Day	Clozapine dose (mg)	Plasma level (ng/mL)	hs‐TnT (ng/L)	CRP (mg/L)	Eosinophils (G/L)
0	0	—	22	1.6	0.63
1	6.25	—	—	—	—
7	12.5	—	20	1.9	0.52
14	25	—	22	1.3	0.52
21	31.25	—	26	2.4	0.62
28	43.75	—	24	4.6	0.66
33	50	30	17	2.4	0.61
47	75	—	—	—	—
70	118.75	56	20	2.0	0.51
84	150	—	—	—	—
92	175	90	17	1.2	0.59
106	218.75	136	28	2.1	0.58
120	262.5	156	28	1.7	0.62
134	300	266	24	1.8	0.64
188	450	435	24	3.4	1.22
202	450	278	24	1.8	0.7

Since initiating clozapine, the patient never developed any clinical symptoms or signs. Weekly laboratory tests never showed any new abnormalities: CRP was always below 5 mg/L; hs‐TnT was always between 17 and 28 ng/L, which means it did not change from the baseline levels and never exceeded the diagnostic threshold of a twofold increase. All other cardiac markers including CK, CK‐MB, and NT‐pro BNP were always within range. The results of the laboratory testing mentioned are summarized in Table 1.

Weekly ECGs also never showed any abnormalities. On Day 104, almost 15 weeks after initiation, a TTE was performed, showing no abnormalities with a normal LVEF of 63%. After titration of clozapine, the patient’s psychiatric condition improved: He showed a marked improvement in his negative symptoms and impulsive‐aggressive behavior, while his psychotic symptoms (grandiose religious delusional beliefs) remained almost unchanged. Due to increased overall stability and higher level of daily functioning, the patient was able to be moved from our high‐security ward to a specialist forensic nursing home 7 months after initiation of clozapine rechallenge. The clozapine dose at discharge was 450 mg. The last serum level, measured 3 weeks prior to discharge, was 278 ng/mL (trough and steady‐state conditions). Comedication at discharge was: amisulpride 600 mg, diazepam 10 mg, ivrabradine 15 mg, bisoprolol 2.5 mg, pantoprazole 20 mg, sodium picosulfate 5 mg, and atropine 0.5% (four drops sublingually).

After moving to the specialist forensic nursing home, the patient quickly adapted to the new environment. Eight months after discharge (15 months after clozapine initiation), the patient was still contentedly living in the nursing home and continuing to take clozapine. His psychopathological condition had stabilized further. While his negative symptoms did not improve after discharge, his delusional beliefs were less prominent and had less impact on his daily actions. He did not exhibit any impulsive‐aggressive behavior. Three months after discharge (10 months after initiating clozapine), a TTE was repeated, showing no abnormalities and a normal LVEF of 60%.

## 3. Discussion

Our case describes a 40‐year‐old male forensic patient diagnosed with paranoid schizophrenia who was successfully rechallenged with clozapine almost 4 years after experiencing CAM.

To our knowledge, there are only 31 published cases of successful clozapine rechallenge after CAM. Our case is the 32nd successful case overall and the 7th successful case that followed a standardized rechallenge protocol. It is the first published and successful case following the instruction recently published by Qubad et al. [6]. Our case is, therefore, a contribution to the development of standardized rechallenge protocols, which could give clinicians and patients more safety in rechallenging clozapine after CAM.

During his first clozapine treatment, from Day 4, the patient experienced tachycardia with some transient episodes of palpitations or chest pain. Otherwise, he did not show any clinical symptoms or signs, especially no fever, low blood pressure, or dyspnea. In addition, his main symptom, tachycardia, is rather unspecific for myocarditis, as it is a fairly common cardiac side effect of clozapine, affecting 25% of patients independently of myocarditis [9]. Apart from this, rather inapparent clinical presentation and an ECG showing no abnormalities, the patient did not show any major laboratory abnormalities: Hs‐TnT was only slightly elevated, never reaching the threshold of a twofold increase. Unfortunately, CRP was never assessed during this period, which was a major mistake probably delaying the diagnosis of CAM. Due to this rather inapparent clinical presentation and scheduling difficulties caused by the ongoing Covid‐19 pandemic, the diagnosis of CAM was delayed by 3 months. The fact that the patient still had echocardiographic abnormalities, including a reduced LVEF, 3 months after clozapine discontinuation suggests that the severity of the myocarditis may have been even greater at onset. The case presented here highlights the difficulties in diagnosing CAM, as its clinical presentation is often described as being rather inapparent. The case is also an example of the relationship between titration speed and the risk of developing CAM: The faster the dose is increased, the higher the risk [6, 15]. A too‐rapid titration can lead to clozapine‐induced inflammation which can inhibit clozapine metabolism and further increase serum levels as part of a positive feedback mechanism [16]. Therefore, the risk of developing CAM can be significantly reduced by choosing a slow tapering rate. Data from different countries using different titration schedules shows that the incidence of CAM is lowest where clozapine is titrated more slowly and personalized, particularly in the Netherlands and Denmark [14]. Implementing slower and personalized titration schedules based on the patient’s ancestry and factors impairing clozapine metabolism (e.g., infections and comedication) while performing baseline and weekly CRP assessments can substantially increase the safety of clozapine use worldwide [14]. In his first treatment with clozapine, the patient reached a dose of 100 mg quite rapidly by Day 4. Compared with international guidelines [10], this titration speed was too fast: For male smokers of European or Western Asian ancestry being treated as inpatients, a clozapine dose of 100 mg should not be reached before the end of the first week. If a patient’s metabolism is even lower (e.g., due to systemic inflammation), a dose 100 mg should not be reached before the third week. The patient’s comedication should have been an additional reason to reduce the titration speed: As the coprescription of quetiapine or olanzapine may increase the risk of CAM, it has recently been recommended that these patients be treated as if they were poor metabolizers [14, 17]. The patient took quetiapine 200 mg throughout the entire titration process. Although olanzapine was stopped on Day 1, it was expected to remain present at relevant concentrations in the blood for at least a few more days, given its mean half‐life of 33 h in healthy individuals [18].

In addition to the fast titration speed, further mistakes were made during the first clozapine treatment: Baseline CRP and hs‐TnT were not assessed, even though the elevated WBC count was suggestive of systemic inflammation. According to international guidelines [10], baseline CRP is a standard assessment recommended to rule out inflammation. Ruling out baseline inflammation is important since cytokines released by inflammation can inhibit the clozapine metabolism (mainly mediated by CYP1A2), thereby increasing clozapine serum levels and the risk of developing CAM [16]. Therefore, if baseline CRP is abnormal, clozapine should not be started until the cause of the inflammation has been identified and resolved [10]. In cases of bacterial or viral infection, the recently published expert consensus recommends waiting at least two to 3 weeks before initiating clozapine treatment [19]. Furthermore, despite being recommended by the aforementioned guidelines, weekly assessments of CRP and hs‐TnT for at least 4 weeks were not performed. If CRP is normal at baseline and becomes abnormal during titration, this is a possible sign for either clozapine‐induced inflammation (CAM being the most common form), which is associated with too‐rapid titration or another concurrent infection [16]. In this case, no assessment of CRP was performed, even after the patient developed clinical signs on Days 4, 11, and 12. When the patient experienced chest pain, CAM should have been more carefully considered and clozapine should have been discontinued until a cardiac evaluation had been performed. In retrospect, this case of CAM occurring during the first challenge likely could have been prevented by following international guidelines [10], which include ruling out baseline inflammation and choosing a slower titration speed. Clozapine would not have been started without first ruling out baseline inflammation.

Fortunately, the patient’s CAM resolved completely. The period between CAM and rechallenge was quite long, at 4 years. McMahon et al. [5] found that a longer period is associated with a higher chance of success. The patient’s clozapine rechallenge was successful. The choice of a very slow titration rate following Qubad et al. [6] resulted in a titration period of 6 months (Table 1), which means that patience and a good therapeutic relationship are needed to bridge the time until clozapine can show its clinical effect. However, it is very important to take the time, as a slower titration speed is associated with a higher success rate [5]. The recently published Delphi‐based consensus also highlights the importance of a slow titration when rechallenging clozapine in patients after CAM [19]. This consensus is even less strict that the protocol used here by Qubad et al. [6], recommending a starting dose of 6.25 mg, followed by a titration of 25 mg per week. Although the patient’s positive symptoms proved resistant to treatment, clozapine resulted in a marked improvement in his negative symptoms and impulsive‐aggressive behavior, leading to increased overall stability. After 2 years of treatment on our high‐security ward, he was able to move to a specialist forensic nursing home.

## 4. Conclusion

This case highlights the potential for a successful clozapine rechallenge in patients with a history of CAM when following a very slow and carefully monitored titration protocol as described by Qubad et al. [6]. Despite the high risks associated with CAM, the patient tolerated the rechallenge without complications, achieving significant improvements in negative symptoms and overall stability. This case supports the growing evidence that, under stringent monitoring and individualized protocols, clozapine rechallenge can be a viable and safe option for patients with TRS.

## Funding

Open access publishing facilitated by Universitat Zurich, as part of the Wiley ‐ Universitat Zurich agreement via the Consortium Of Swiss Academic Libraries.

## Consent

The patient gave his written consent that his medical history could be used to create this case report. Additionally, personally identifiable details have been removed whenever possible.

## Conflicts of Interest

The authors declare no conflicts of interest.

## Data Availability

The data that support the findings of this study are available upon request from the corresponding author. The data are not publicly available due to privacy or ethical restrictions.
